# The Contribution of *α*-Synuclein Spreading to Parkinson's Disease Synaptopathy

**DOI:** 10.1155/2017/5012129

**Published:** 2017-01-03

**Authors:** Francesca Longhena, Gaia Faustini, Cristina Missale, Marina Pizzi, PierFranco Spano, Arianna Bellucci

**Affiliations:** ^1^Department of Molecular and Translational Medicine, University of Brescia, Brescia, Italy; ^2^IRCCS Fondazione Ospedale San Camillo (NHS-Italy), Venice Lido, Italy

## Abstract

Synaptopathies are diseases with synapse defects as shared pathogenic features, encompassing neurodegenerative disorders such as Parkinson's disease (PD). In sporadic PD, the most common age-related neurodegenerative movement disorder, nigrostriatal dopaminergic deficits are responsible for the onset of motor symptoms that have been related to *α*-synuclein deposition at synaptic sites. Indeed, *α*-synuclein accumulation can impair synaptic dopamine release and induces the death of nigrostriatal neurons. While in physiological conditions the protein can interact with and modulate synaptic vesicle proteins and membranes, numerous experimental evidences have confirmed that its pathological aggregation can compromise correct neuronal functioning. In addition, recent findings indicate that *α*-synuclein pathology spreads into the brain and can affect the peripheral autonomic and somatic nervous system. Indeed, monomeric, oligomeric, and fibrillary *α*-synuclein can move from cell to cell and can trigger the aggregation of the endogenous protein in recipient neurons. This novel “prion-like” behavior could further contribute to synaptic failure in PD and other synucleinopathies. This review describes the major findings supporting the occurrence of *α*-synuclein pathology propagation in PD and discusses how this phenomenon could induce or contribute to synaptic injury and degeneration.

## 1. Introduction

Pathological accumulation of *α*-synuclein in the brain is a typical neuropathological hallmark of Parkinson's disease (PD), a complex neurodegenerative disorder characterized by motor disability that derives from the neurodegeneration of nigrostriatal dopaminergic neurons. The presence of Lewy bodies (LB) and Lewy neurites (LN), proteinaceous inclusions whose main constituent is fibrillary-aggregated *α*-synuclein, is a defining neuropathological alteration observed in the brain of affected patients. In the last few years, it has become evident that PD may be considered as a synaptopathy [1, 2]. Indeed, striatal dopaminergic terminal loss appears to precede neurodegeneration in the substantia nigra [3, 4] and the deposition of *α*-synuclein, which is considered as a causative factor for the onset of the disorder, mainly affects synaptic terminals in its early stages [5, 6]. Nonetheless, the exact molecular mechanisms that determine the selective vulnerability of nigrostriatal synapses to *α*-synuclein deposition are still enigmatic. One of the reasons for the reduced resilience of dopaminergic terminals to *α*-synuclein accumulation may be the fact that dopamine neurons of the substantia nigra show relatively large axonal lengths with 10–100 times the number of synapses of neighboring neuronal cells [7]. This implies that these cells require an elevated energy demand to efficiently sustain the trafficking of organelles and vesicles to allow proper synapse functioning [8]. Hence, they might result particularly susceptible even to subtle homeostatic changes at synaptic sites, which constitute their major source of energy consumption.

Compelling evidence indicates that *α*-synuclein can spread from diseased to healthy cells, thus contributing to disease worsening [9]. Indeed, graft-derived dopamine neurons can develop LB pathology several years after transplantation [10–12], an event that can be responsible for the loss of beneficial effects of cell therapy along with time. In addition, both neurons and astrocytes have been found to internalize disease-associated *α*-synuclein in the postmortem brain of subjects with Lewy pathology, suggesting prion-like spread of *α*-synuclein by uptake from surrounding structures [13].

It is worth noting that the progression of PD symptoms seems to correlate with the topographical brain propagation of *α*-synuclein pathology between anatomically interconnected brain areas [14, 15], suggesting the occurrence of trans-synaptic spreading of pathology. This is not surprising when considering that *α*-synuclein is a synaptic-vesicle associated protein [16]. Studies on experimental models of PD have confirmed the occurrence of trans-synaptic transmission of pathological *α*-synuclein* in vivo* [17–20]. In light of evidences supporting host-to-graft *α*-synuclein passage, as well as the ability of the protein to propagate protein misfolding in recipient cells, a “prion-like” hypothesis of PD has been postulated [21–29]. Indeed, prions are transmissible misfolded conformers of the prion protein, PrP, which seed further generation of infectious proteins [30]. However, the mechanisms mediating *α*-synuclein release, uptake, and behavior in recipient cells deserve further investigation to definitely assert that *α*-synuclein behaves like a prion. For instance, not all the postmortem analyses on PD cases seem to confirm the caudo/rostral spread of *α*-synuclein pathology throughout the brain described by Braak [14, 15]. Moreover, the ability to seed aggregation in recipient cells has been found to be dependent on high concentration of aggregates in the face of the need of low amount of aggregates to induce cytotoxicity [31]. These evidences pose into question both the trans-synaptic spreading hypothesis and the prion-like behavior of *α*-synuclein. Nonetheless, cell-to-cell transmission of *α*-synuclein can occur with Ca^2+^-dependent exosome-mediated release [32] or nonclassical exocytosis [33] as the most plausible candidate mechanisms for *α*-synuclein ejection. Finally, what could be the transmissible form of the protein that can mediate toxicity and/or function as seed for “misfolding” propagation in recipient cells between monomeric, oligomeric, and fibrillary *α*-synuclein still needs to be clarified. In this review, we describe the features of *α*-synuclein pathology spreading in PD and discuss how this could contribute to synaptic damage.

## 2. Alpha-Synuclein Function at the Dopamine Synapse

Αlpha-synuclein was first identified in the synaptic vesicles and nuclei of the electric organ of* Torpedo Californica *[34]. The protein is highly expressed in presynaptic terminals of the brain and peripheral nervous system, where it associates with the synaptic vesicle apparatus [35–37]. The expression of *α*-synuclein is elevated within the synapses of nigral dopamine neurons [38]. There, the protein can modulate synaptic dopamine release by directly modulating the dopamine transporter (DAT), synapsin III, the small GTP-binding protein Rab3A, and the soluble N-ethylmaleimide sensitive fusion attachment protein receptor (SNARE) protein member vesicle associated membrane protein-2 (VAMP-2) [6]. Indeed, it catalyzes the entry of VAMP-2 into the SNARE complex [39] and enhances DAT localization at the plasma membrane [40], which consistently is impaired by *α*-synuclein aggregation [41, 42]. The toxicity exerted by *α*-synuclein deposition in dopaminergic neurons is rescued by Rab3A expression, suggesting that this protein is relevant for its normal function [43]. Conversely, *α*-synuclein membrane association is regulated by the Rab3A recycling machinery and presynaptic activity [44]. Finally, synapsin III distribution and expression in dopaminergic neurons is regulated by *α*-synuclein, while synapsin III gene silencing inhibits *α*-synuclein aggregation [4].

**Figure 1:**
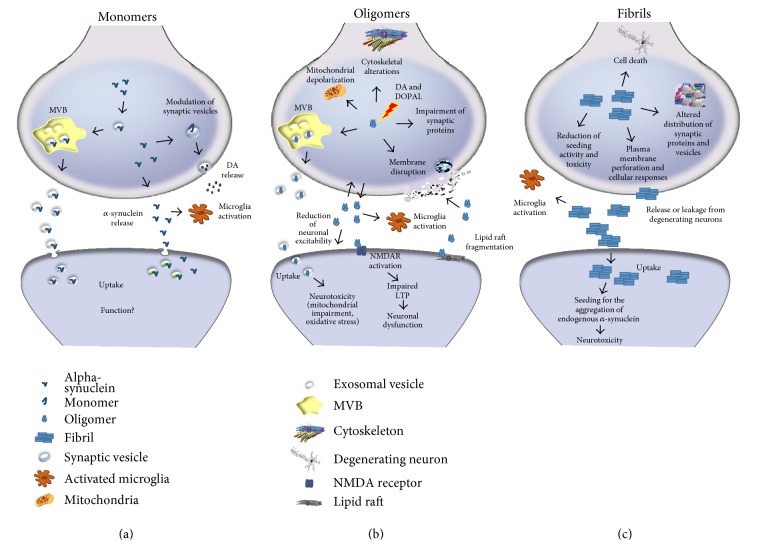
Monomeric, oligomeric, and fibrillary *α*-synuclein at the synaptic terminal. (a) Monomeric *α*-synuclein modulates synaptic function by controlling synaptic vesicle release. This form of the protein can be released in association with exosomes, activates microglial cells, and can be internalized at postsynaptic sites. (b) Oligomeric *α*-synuclein formation is enhanced by interaction of monomeric protein with DA. Alpha-synuclein oligomers can form a stable adduct with the toxic dopamine metabolite DOPAL. Oligomers can be released in association with extracellular vesicles and then activate microglia. Alpha-synuclein oligomers can disrupt synaptic vesicles membranes as well as presynaptic and postsynaptic membranes. Exogenous *α*-synuclein oligomers can damage lipid rafts and affect LTP by activating NMDA receptors. Intracellular *α*-synuclein oligomers with endogenous or exogenous origin impair mitochondrial functions and cytoskeletal architecture. (c) Fibrillary-aggregated *α*-synuclein alters synaptic vesicle release by clustering synaptic vesicles and by perforating plasma membrane. Extracellular fibrils deriving from degenerating neurons in the PD brain can activate microglial cells and actively contribute to alpha-synuclein pathology spreading. The formation of endogenous *α*-synuclein fibrils can reduce seeding activity and toxicity although exogenous *α*-synuclein fibrils function as a seed for the aggregation of endogenous *α*-synuclein in recipient cells.

In the synapse, likewise in the other neuronal compartments, *α*-synuclein exists in a dynamic equilibrium between a soluble state and a membrane-bound state, with its affinity for synaptic vesicles being higher than that for cell membranes [45–47] (Figure 1(a)). However, when interacting with cell membranes, the protein presents higher affinity for lipid rafts [48]. The interaction between *α*-synuclein and lipid membranes is also relevant for the protein to exert its functions [49]. This, notwithstanding, *α*-synuclein presents elevated structural plasticity and its effective conformation in soluble and membrane-bound state is matter of debate. While some evidences support the existence of a stable cytosolic tetrameric form of the protein, other studies have shown that it can be found as a disordered monomer in the central nervous system (CNS) and other mammalian cells or that these forms both coexist in a dynamic equilibrium [50, 51]. More recently, the existence of a stable dimer has been suggested by computational evidences [52]. As for the aminoacidic sites involved in the interaction with membranes, numerous studies have reported a key role for the ones located at the N-terminal portion of *α*-synuclein [53]. Jiang and coauthors [54], by coupling neutron reflectometry (NR) and fluorescence spectroscopy analysis, have found that the N- and C-terminal regions near positions 4 and 94 are anchored to the membrane, while the putative linker spanning residue 39 samples multiple conformations, which are sensitive to the chemical nature of the membrane surface. The mechanism of *α*-synuclein binding to lipid membranes has been found to be primarily dependent on the surface charge density of the lipid bilayer and the phase state of the lipids, with *α*-synuclein possessing lipid ordering effect and thermally stabilizing vesicles [55]. These findings suggest that this process might be tunable by environmental changes.

Collectively, these evidences support that subtle changes in *α*-synuclein structural folding, likewise the formation of oligomers or insoluble aggregates, can severely compromise the activity of dopaminergic neurons, with the adjunct of cell-to-cell transmission likely worsening and perpetrating the related injury.

## 3. Central and Peripheral Localization of Neuronal *α*-Synuclein Pathology in PD: Trans-Synaptic or Systemic Spreading?

The presence of different forms of *α*-synuclein in cerebrospinal fluid (CSF), blood plasma [56–58], and saliva [59] coupled to the discovery of *α*-synuclein pathology in embryonic nigral transplants of PD patients [10, 11, 60] suggested that *α*-synuclein can move from cell to cell and can initiate pathology in recipient neurons. It is well established that *α*-synuclein can be secreted [61, 62], a process that is thought to be mainly mediated by exosomal vesicles [63, 64].

Compelling evidence indicates that in PD *α*-synuclein pathology is not confined within the brain. LB-like aggregates of the protein have been found in the dorsal root ganglia, as well as in several tracts of the peripheral nervous system such as gastrointestinal innervation, motor nerves innervating the pharyngeal muscles, cranial and spinal nerves, skin nerves, and olfactory epithelium [8, 65–70]. The presence of these peripheral aggregates has been proposed to associate with typical PD premotor symptoms [71] and could contribute to disease onset in the brain, as supported by evidences indicating that vagotomy diminishes the risk to develop the disorder [20]. As hypothesized by Braak and colleagues [14], caudo-rostral spreading of *α*-synuclein has been found to occur through vagal connections in experimental models of PD [17]. Resection of the vagal autonomic projection impedes the peripheral-to-CNS diffusion of pathological *α*-synuclein and the onset of PD-like motor phenotype in the chronic intragastric rotenone model of PD [19], supporting a causative role for protein spreading in the onset of the disorder. Vagotomy eliminates most, but not all, *α*-synuclein-positive neurites in the plexus, thus providing a candidate pathway for the retrograde transport of putative PD pathogens or toxins from the enteric nervous system to the central nervous system [67]. However, even vagal nerve impairment per se has been found to induce dopamine functional damage, therefore suggesting that the vagal degeneration occurring in the PD brain [72, 73] might be pivotally involved in PD pathogenesis independently from *α*-synuclein pathological spreading. Studies in rodents have shown that vagal afferent endings in the myenteric plexus and the gastrointestinal smooth muscle do not express *α*-synuclein, whereas virtually all vagal preganglionic projections to the gut show *α*-synuclein immunopositivity both in axons and in terminal varicosities in apposition with myenteric neurons.

However, some other studies raise some concerns about its validity as *α*-synuclein accumulation within the peripheral nervous system can occur also in neurologically intact subjects with aging [74]. Not by chance, PD and aging have been proposed to be a unique entity and the disease has been postulated to manifest in all subjects whether they could live long enough [75]. In addition, typical manifestations of aging such as frailty that interestingly has been found to associate with brain neuropathological accumulation of LB and nigral neuronal loss [76, 77] also characterize PD [78]. Other studies, failing to detect either the typical pattern of topographical distribution of *α*-synuclein pathology in the postmortem brain of affected patients [79, 80] or the development of pathology spreading following preformed fibril-inoculation* in vivo*, cast further confusion over the prion-like hypothesis.

Recent studies indicate that *α*-synuclein oligomers are increased in red blood cells and CSF of PD subjects and could serve as biomarkers of disease [81, 82]. The levels of *α*-synuclein are also increased in peripheral lymphocytes [83] as well as in plasma and CNS-derived exosomes of affected individuals [84]. The systemic spreading of the protein could thus also involve exosomal-mediated release and biological fluids. Indeed exosomes are small membrane vesicles which result from exocytosis of multivesicular bodies. They function as mediators of intercellular communication, as they transfer specific proteins, lipids, microRNAs, and DNA between cells. Because of their small size, exosomes can move from the site of discharge by diffusion and reach several biological fluids, such as blood, CSF, urine, and synovial fluid [85]. Consistently, plasma exosomal *α*-synuclein is likely CNS-derived and increased in PD [84] and CSF exosomes have been found to induce *α*-synuclein aggregate formation in recipient healthy cells [86]. This suggests that the circulatory system could also mediate *α*-synuclein systemic spreading, with the choroid plexus being likely involved in the passage of alpha-synuclein from the blood to the brain and vice versa. Notably, the presence of increased *α*-synuclein immunoreactivity at this site has been described in PD [13]. However, it still remains to be determined whether a link effectively exists between peripheral and brain *α*-synuclein deposition. In the case that they might constitute a unique phenomenological entity, defining what might come first between central and peripheral deposition could be crucial for determining the causes of PD.

## 4. Toxicity of *α*-Synuclein Oligomers and Fibrils: A Still Unresolved Dichotomy

Aggregation of *α*-synuclein is a critical step in the etiology of PD, with prefibrillar oligomers of the protein that might constitute the direct precursors of fibrils being involved in neurodegenerative process [87, 88]. Even if the injection of fibrils into the rat brain is found to be more toxic than that of oligomers and ribbons, as it induces neurodegeneration and motor impairment, all these species can self-amplify* in vivo* and lead to PD/multiple system atrophy-like alterations in the injected animals [89]. This suggests that strain-specific prion-like infectivity and symptomatology characterize different *α*-synuclein conformers, whose biochemical nature is still unknown. Therefore, asserting which, between fibrils or oligomers, are the most toxic species is still difficult at present. Nonetheless, the analysis of both oligomers' and fibrils' structure could help to elucidate this conundrum. In addition, data supporting that monomeric, oligomeric, and fibrillary *α*-synuclein can activate microglial cells [90–93] suggest that all these forms of the protein could affect neuronal homeostasis by modulating microglia function that could be either protective or detrimental in PD [94].

As for oligomers, those formed by a peptide derived from residues 36–55 of *α*-synuclein were recently characterized by X-ray crystallography [95]. The authors showed that this specific peptide is able to adopt a *β*-hairpin structure, which assembles in a hierarchical fashion. Three *β*-hairpins then assemble to form a triangular trimer and three copies of the triangular trimer then assemble to form a basket-shaped nonamer that couple to form an octadecamer. Following molecular modeling analysis, these authors also proposed that full-length *α*-synuclein might be able to assemble in this fashion. Circular dichroism spectroscopy demonstrated that the peptide 36–55 interacts with anionic lipid bilayer membranes, like oligomers of full-length *α*-synuclein, and is found to be toxic in neuronal cell models. Other cryoelectron microscopy studies have shown that oligomers isolated during fibril formation possess a hollow cylindrical architecture with similarities to certain types of amyloid fibril [96].

The formation of *α*-synuclein dimers has been described to initiate aggregation and neurotoxicity [97]. Computer modeling and cell-based studies have revealed that upon interaction with plasma membranes *α*-synuclein rapidly penetrates them, changing its conformation from *α*-helical toward a coiled structure. This penetration facilitates the incorporation of additional *α*-synuclein monomers to the complex and subsequent displacement of phospholipids and formation of oligomers in the membrane [98]. Consistently, *α*-synuclein oligomers neurotoxicity* in vivo* has been found to be mediated by membrane disruption [99].

Chen and coauthors described that stable toxic *α*-synuclein oligomeric species with a hollow cylindrical architecture with similarities to certain types of amyloid fibril can be trapped during fibril formation [96]. Their study showed that the *β*-sheet geometry acquired in the early stages of the self-assembly process plays a key role in dictating the kinetic stability and the pathological nature of individual oligomeric species. Spectroscopy studies have also shown that whereas fibrils adopt a parallel arrangement oligomers adopt an antiparallel *β*-sheet structure [100], thus suggesting that differences in the toxicity of these species might rely on their diverse conformations. The neurotoxicity of oligomers has been demonstrated in different experimental conditions. Alpha-synuclein overexpression in neuroblastoma cells causes the formation of *α*-synuclein oligomeric species, whose presence is associated with mitochondrial fragmentation and autophagic-lysosomal pathway activation in live cells [101]. The accumulation of oligomeric and fibrillar forms of *α*-synuclein has a negative impact on mitochondria function by inhibiting mitochondria complexes IV and V in the striatum [102], reducing mitochondrial Ca^2+^ release and NADH oxidation [103]. Permeabilization of mitochondria membranes can also be induced by *α*-synuclein oligomers [104–107]. The accumulation of toxic *α*-synuclein oligomers in the endoplasmic reticulum (ER) has been described as a feature of *α*-synucleinopathies [108, 109]. Recently, *α*-synuclein oligomers have been found to interact with metal ions to induce oxidative stress and neuronal death in PD [110]. Interestingly, the oligomer-induced reactive oxygen species (ROS) production was independent of several known cellular enzymatic sources and relied solely on the presence of free metal ions.

Studies on human *α*-synuclein transgenic mice have shown that the accumulation of oligomers mainly occurs at synaptic sites and is crucial for the induction of synaptic and neuronal degeneration [111]. Consistently, the formation of *α*-synuclein soluble oligomers can reduce neuronal excitability of neocortical pyramidal cells, suggesting that it could impact on network activity [112]. Oligomers have been found to affect synaptic function by changing lipid raft composition and increasing N-methyl-D-aspartate (NMDA) receptor activation at postsynaptic membranes [113, 114].

Alpha-synuclein oligomers could thus easily impinge on synaptic resilience by disrupting membranes, inducing mitochondrial depolarization, altering cytoskeleton dynamics, impairing protein clearance pathways, and enhancing oxidative stress [115] (Figure 1(b)). In addition, the preferential degeneration of dopaminergic neurons in PD [116, 117] led to the hypothesis that dopamine may play an important role in *α*-synuclein oligomerization [118]. The oxide forms of dopamine, generated by oxidative stress, accelerate formation of *α*-synuclein oligomers as an endogenous protofibril stabilizer, demonstrating the connection between dopamine and *α*-synuclein oligomer formation [119, 120]. The toxic dopamine metabolite 3,4-dihydroxyphenylacetaldehyde (DOPAL) can compromise the membrane-binding affinity of *α*-synuclein to synaptic vesicles, as well as its fibrillation, by forming an adduct with the protein, thus reducing its functional ability to modulate synaptic vesicles [121].

Fibrils exert their toxic actions by activating other detrimental processes in neuronal cells. This may be related to the different conformation of oligomers and fibrils. Consistently, Curtain and coauthors [122], by using small angle X-ray scattering and ensemble optimization modeling studies, were able to demonstrate that *α*-synuclein oligomers and fibrils originate in two distinct conformer pools, with E53T and E45K mutations enhancing the tendency to form fibrils, while the A30P conferring propensity toward oligomer assembly. This is in line with previous data reporting that A30P mutant *α*-synuclein forms different fibril structures [123] and that mutations in the KTKE(Q)GV imperfect amino acid repeats in the N-terminal part of the protein can also affect its tendency toward fibril formation [124]. Mutant *α*-synuclein preferentially shifts from monomer to fibrils, thus suggesting that the formation of these species plays a crucial role in the pathophysiology of early-onset PD [125, 126]. Whether the lipid bound *α*-helical form or the unfolded state initiates protein aggregation remains to be determined.

X-ray and electron diffraction of the *α*-synuclein aggregates showed that the fibrils consist of a *β*-sheet structure in which the *β*-strands run perpendicularly to the long fiber axis [127]. A flexible break close to residues 52–55 has been found to be relevant for fibril formation [128], while the negative charges and aromatic residues at the C-terminal region play a modulatory role on fibrillation [129, 130]. In addition, molecular dynamics simulations allowed observing that residues 36−55 of the nonamyloid component (NAC) domain, in the central region of the protein, are important for the formation of *β*-hairpin and that the point mutations stabilize this *β*-hairpin that represented the first step of *α*-synuclein aggregation [95].

Αlpha-synuclein fibrils can exert toxicity by disrupting the normal distribution of membrane proteins and synaptic vesicles [6], perforating plasma membranes [131, 132], triggering cellular responses [133], and causing death in cell systems [134] (Figure 1(c)). However, the stabilization of fibril clusters can prevent fragmentation and reduces seeding activity and toxicity [135]. In addition, *α*-synuclein structure is strictly dependent on membrane interactions that can both accelerate [136, 137] and inhibit [136, 138, 139] fibril formation.

## 5. Mechanisms of *α*-Synuclein Release

Since 2003, Braak and colleagues had speculated that, in idiopathic PD, LB pathology could spread from the enteric nervous system or the olfactory bulb to precise brain regions during the progression of the disease [14], thus suggesting the occurrence of cell-to-cell *α*-synuclein transmission. The existence of this phenomenon was confirmed thanks to postmortem studies carried out on the brain of PD patients who had received transplants of mesencephalic neurons 14 years prior to the demise. Indeed, the analysis of autopsy samples showed that grafted neurons had developed LB pathology [10, 60]. Host-to-graft transmission was then corroborated in different experimental models* in vivo* [140–142].

The first evidence in favor of *α*-synuclein release was provided by Dixon and colleagues that demonstrated that, in yeasts, the protein can be delivered to the extracellular space through the classical ER-to-Golgi secretory pathway [143]. However, these findings were not confirmed in SH-SY5Y cells, where *α*-synuclein has been observed to be mediated by ER/Golgi-independent unconventional exocytosis pathway [33, 62] and in a Ca^2+^-dependent manner by exosomes [63], respectively. Protein overexpression, misfolding, and posttranslational modifications can promote *α*-synuclein accumulation and release, caused by impaired intracellular degradation. Several cellular mechanisms have been found to mediate *α*-synuclein degradation, including the ubiquitin-proteasome system (UPS) and lysosome-mediated digestion [144]. In PD, dysfunction of the UPS causes the accumulation of misfolded *α*-synuclein and is thought to be highly implicated in the pathogenesis of PD [145–149]. However, ubiquitination of *α*-synuclein in LB has been found to constitute a pathological event not associated with impairment of proteasomal function [150]. Alpha-synuclein can also be cleared by the autophagy-lysosome pathway (ALP). Indeed, Cuervo [151] showed that wild type *α*-synuclein is translocated into lysosome for degradation. Conversely, aggregated *α*-synuclein can cause a malfunction in lysosomal degradation pathway [152]. Remarkably, also aging may represent another important factor related to the dysfunction of protein control systems [145, 151, 153–155] causing a vicious circle that exacerbate the accumulation of toxic forms of *α*-synuclein.

The oxidized forms of *α*-synuclein are preferentially secreted [62] and studies in induced pluripotent stem cell-derived neurons harboring *α*-synuclein gene locus triplications showed that increased intracellular levels of the protein foster its release [156]. However, the amount of released *α*-synuclein deriving from injured neurons is likely very low as neuronal damage and neurodegeneration do not exacerbate its propagation [18].

At the subcellular level, it has been observed that, in neurons, *α*-synuclein can be found in the three endosomal compartments and translocate across them (early, late, and recycling endosomes) [32]. This pathway could be involved in the process of protein release [32]. Moreover, treatments that could disturb the homeostasis of the endosomal system alter *α*-synuclein spreading [63, 157]. From the early endosomes, a portion of the protein can be translocated to the recycling endosomal compartment, which can fuse with the plasma membrane to allow the release of soluble forms of *α*-synuclein. This process seems to be mediated by the interaction between *α*-synuclein and Rab GTPases [158]. The portion of protein that remains in the endosomal compartment is targeted to multivesicular bodies (MVBs), which usually fuse with lysosomes for protein degradation. In diseased neurons, where the accumulation of toxic *α*-synuclein aggregates could perturb the activity of protein clearance mechanisms, MBVs can release the intraluminal vesicles in the extracellular space as exosomes [159]. Remarkably, numerous findings [63, 157, 160, 161] support that *α*-synuclein can be released in association with exosomes, small vesicles (40–100 nm in diameter) originating from the endosomal compartment. Exosomes can be secreted and interact with cell membranes in a cell type-dependent manner and recipient cells can internalize them through different endocytic mechanisms [162]. In several neurodegenerative diseases, exosomes seem to play the role of “garbage” carrier, acting as an alternative pathway of protein elimination when intracellular mechanisms are engulfed [163, 164]. The process through which *α*-synuclein is targeted to the endosomal compartment is still matter of study, with a recent work suggesting posttranslational modifications of the protein being involved. In particular, the conjugation of Small Ubiquitin like Modifier (SUMO) also defined as SUMOylation of *α*-synuclein triggers its internalization in exosomes [160]. To date, SUMOylation serves to regulate the solubility of aggregation-prone proteins [165, 166], as well as a ubiquitin-independent endosomal sorting complex required for transport (ESCRT) sorting signal, regulating the extracellular vesicle release of *α*-synuclein [160]. Interestingly, ubiquitinated forms of *α*-synuclein have been found to be present in LB [167, 168] and the ubiquitination of the protein has been found to increase its aggregation propensity and neurotoxicity* in vitro* [169, 170]. Taken together, these findings suggest that *α*-synuclein ubiquitination and SUMOylation may be coexisting phenomena whose equilibrium regulates the folding state and the localization of the protein. In particular, the fact that SUMOylation facilitates the exosomal-mediated release of *α*-synuclein [160] supports the hypothesis that exosomes might behave as possible “way out” to eliminate exceeding amount of the protein that cannot be degraded by conventional mechanisms such as proteasome or lysosome digestion. Further studies are needed to corroborate the role of exosomes in toxic *α*-synuclein spreading and in the progression of PD. Nonetheless, exosomes and their cell-to-cell transmission mechanisms could represent novel intriguing therapeutic targets to lessen or block the evolution of *α*-synuclein pathology.

## 6. Uptake of Aberrant *α*-Synuclein from Recipient Cells: Consequences on Synaptic Functions

In the brain, neurons can show differential vulnerability to *α*-synuclein accumulation. Indeed, even though it has been reported that in PD subjects LB pathology first affects the vagal nuclei, locus coeruleus, and olfactory bulbs, the motor symptoms appear when *α*-synuclein deposition reaches nigrostriatal dopamine neurons. In particular, since the striatal synaptic accumulation of the protein is much higher than its deposition in the cell bodies of the substantia nigra [171], it may be hypothesized that mechanisms promoting its synaptic translocation could be enhanced in the early phases of disease. When taking into account the trans-synaptic spreading hypothesis by Braak, it is feasible that nigrostriatal dopamine neurons might interpret the uptake of *α*-synuclein pathological seeds as a signal that prompts them to further promote *α*-synuclein trans-synaptic transfer as a response. In line with this idea, the injection of synthetic fibrils and LB extracts from PD subjects in the mouse brain has been found to induce a rapid and progressive synucleinopathy between anatomically interconnected brain regions [9, 164, 172–174]. Nonetheless, other studies have reported considerable difficulties in inducing a widespread induction of *α*-synuclein pathology following intracerebral administration of amyloidogenic forms of the protein in mice [175]. Furthermore, several mechanisms, such as neuroinflammation, have been found to act synergistically or independently to promote the spread of pathology following fibrillary amyloidogenic and nonamyloidogenic *α*-synuclein [175]. This evidence calls into question that extracellular *α*-synuclein can catalyze aggregation and spread of intracellular protein only through a nucleation dependent conformational templating mechanism. Worthy of note, besides the fact that fragmented amyloid-like aggregates of short *α*-synuclein fibrils can function as seeds that trigger prion-like conversion [176], the transmission of mature fibrils between cells might be difficult in light of their dimensions and stability. It is easier to speculate that they might derive from degenerating neurons in the parenchyma. On this line, brain propagation of *α*-synuclein has been found to involve nonfibrillar protein species and to be enhanced in *α*-synuclein null mice [177]. In addition, PD-causing *α*-synuclein missense mutations shift native tetramers to monomers as a mechanism for disease initiation [51]. Extracellular oligomeric species that are also transmissible through exosomal vesicles [178] are highly abundant in the PD brain [115] and can promote *α*-synuclein aggregation in recipient cells [179]. Transmitted electron microscopy studies in the postmortem human brain of subjects affected by synucleinopathies, reporting the presence of oligomeric *α*-synuclein within the early-endosomal compartment of neuronal cells, are also in line with the idea that oligomers might be the transmissible species [13]. However, what could be the cause of their increased accumulation and how they trigger aggregation of endogenous *α*-synuclein in recipient cells still needs elucidation. The release of oligomers might serve to eliminate exceeding levels of the protein, or it could depend on the activation of plastic structural adaptive mechanisms in neurons as the protein is involved in synaptic plasticity [180, 181]. Otherwise, protein oligomers could behave as a transmissible neuronal messenger between neighboring neurons. Remarkably, the higher stability of *α*-synuclein oligomers and fibrils renders these species more suitable to be secreted in the extracellular space when compared to monomeric protein. Indeed, the conformation of this latter could be modulated even by subtle homeostatic changes in the microenvironment.

Alpha-synuclein uptake could very well contribute to synaptic damage. Indeed, the protein could accumulate at synapses by altering the function of endogenous proteins and engulfing the retrograde transport from the terminals that receive *α*-synuclein to the cell bodies. Alternatively, recipient cells might collapse as they fail to degrade internalized *α*-synuclein efficiently. It is feasible that the accumulation of extracellular *α*-synuclein in dopaminergic synaptic terminals can easily initiate synaptic failure given the relevance of the protein in the regulation of dopamine release [182]. Folding and misfolding of endogenous *α*-synuclein can be modulated by exogenous pathological *α*-synuclein forms and then affect, or be affected by, their interaction with lipid membranes [183]. Alpha-synuclein binding to lipid membranes can be either detrimental or protective to neuronal cells [184]. For instance, A30P, E46K, and A53T disease variants of *α*-synuclein show increased lipid binding affinity [185] although they have distinct membrane permeabilization properties [186] and can thus differentially affect membrane structure [187]. In line with this idea, exogenous *α*-synuclein has been found to induce lipid raft fragmentation thus leading to both pre- and postsynaptic alterations [114] and *α*-synuclein oligomers can impair long term potentiation (LTP) and impair synaptic transmission [113].

Finally, a recent study showed that different *α*-synuclein conformers can cross the blood brain barrier and distribute to the CNS after intravenous injection [89]. This novel evidence suggests that the diffusion of *α*-synuclein pathology might also be mediated by mechanisms other than the simple trans-synaptic spreading of the protein among interconnected brain regions.

Looking at the mechanisms of *α*-synuclein uptake, while the soluble monomeric protein can cross the plasma membrane or can be captured by Rab5a-dependent [188] and dynamin-dependent endocytosis [140], high order assembly can enter into recipient cells by using different endocytic pathways [169]. Among the possible mechanisms of uptake, macropinocytosis has also been explored [189]. Other authors reported that extracellular *α*-synuclein uptake by microglial cells is mediated by the GM1 ganglioside as well as by hitherto-unknown protein receptors in clathrin-, caveolae-, and dynamin-independent, but lipid raft-dependent processes [90]. However, the caveolae-specific protein caveolin-1 has been found to interact with and to mediate *α*-synuclein toxicity in neuroblastoma cells [190] thus suggesting that the possibility that the uptake of the protein might be mediated by caveolae-mediated endocytosis at least in neuronal cells deserves further investigation. More recently, mesenchymal stem cells were identified as blockers of the clathrin-mediated endocytosis of extracellular *α*-synuclein, a process that is controlled by the interaction with NMDA receptor [191]. In addition, the interaction between preformed *α*-synuclein fibrils and immune receptor lymphocyte activation gene 3 (LAG3) has been found to be essential for initiating the transneuronal propagation of *α*-synuclein [192]. This study clearly opens new avenues for PD therapy as LAG3 antibodies are already being tested as cancer treatments [193] and suggested that LAG3 might mediate both immune system activation and systemic spreading of pathological fibrillary *α*-synuclein species.

The fact that *α*-synuclein can be released in association with exosomes and extracellular vesicles (EV) strongly suggests that these might constitute the primary vehicles of cell-to-cell transmission of the protein, preserving it from degradation by extracellular enzymes and facilitating its correct targeting toward recipient cells. Indeed, given the high structural instability of the protein and its small size, it is quite unlikely that *α*-synuclein could easily survive in the brain parenchyma environment in a free and soluble form.

Collectively, these evidences strongly support that *α*-synuclein spreading could very well contribute to synaptic impairment in PD although the biological factors determining the selective vulnerability of nigrostriatal neurons, even on top of the “prion-like” hypothesis, are not yet clear.

## 7. Concluding Remarks

At present, the spreading hypothesis of *α*-synuclein pathology is still matter of debate. Indeed, although a plethora of research studies seem to confirm that *α*-synuclein can diffuse throughout the nervous system, is transmitted from cell to cell, and could induce toxicity and function as a seed for the aggregation of endogenous protein, other evidences seem to confute these findings (Table 1). This, notwithstanding, data on the transmissibility of the protein as well as on the misfolding-inducing ability of *α*-synuclein oligomers and fibrils suggested that these features might easily perturb synaptic homeostasis, especially in dopamine neurons. It could be feasible that *α*-synuclein release, coupled to fibrillary insoluble inclusion formation, could deprive dopamine synaptic terminals from the modulatory action of the protein. In parallel, the exosome-mediated exchange of *α*-synuclein oligomers between neighboring terminals could overwhelm intracellular trafficking by encumbering on the endosomal system. Much work still needs to be done to define the contribution of *α*-synuclein spreading to PD synaptopathy. However, the determination of what can be considered as a transmissible pathological form of *α*-synuclein as well as the mechanisms through which this entity can be transmitted from a diseased presynaptic terminal to a healthy postsynaptic ending can help us to understand much more on PD neurobiology and to identify novel effective therapeutic strategies to cure this disorder. Indeed, whether PD is primarily a disorder of the synapse, novel effective therapeutic approaches should both heal diseased synapses and block the cell-to-cell transmission of toxic *α*-synuclein species.

## Figures and Tables

**Table 1:** 

First author	Title	Year	Journal
	*Evidences supporting α-synuclein spreading*		
Braak	“Staging of Brain Pathology Related to Sporadic Parkinson's Disease”	2003	Neurobiol. Aging
Del Tredici	“Sporadic Parkinson's Disease: Development and Distribution of Alpha-Synuclein Pathology”	2016	Neuropathol. Appl. Neurobiol.
Iljina	“Kinetic Model of the Aggregation of Alpha-Synuclein Provides Insights into Prion-Like Spreading”	2016	Proc. Natl. Acad. Sci. USA
Oh	“Mesenchymal Stem Cells Inhibit Transmission of *α*-Synuclein by Modulating Clathrin-Mediated Endocytosis in a Parkinsonian Model”	2016	Cell Rep.
Helwig	“Brain Propagation of Transduced Alpha-Synuclein Involves Non-Fibrillar Protein Species and Is Enhanced in Alpha-Synuclein Null Mice”	2016	Brain
Bernis	“Prion-Like Propagation of Human Brain-Derived Alpha-Synuclein in Transgenic Mice Expressing Human Wild-Type Alpha-Synuclein”	2015	Acta Neuropathol. Commun.
Illes-Toth	“Distinct Higher-Order Alpha-Synuclein Oligomers Induce Intracellular Aggregation”	2015	Biochem. J.
Ulusoy	“Neuron-to-Neuron *α*-Synuclein Propagation *In Vivo* Is Independent of Neuronal Injury”	2015	Acta Neuropathol. Commun.
Stuendl	“Induction of *α*-Synuclein Aggregate Formation by CSF Exosomes from Patients with Parkinson's Disease and Dementia with Lewy Bodies”	2016	Brain
Prusiner	“Evidence for *α*-Synuclein Prions Causing Multiple System Atrophy in Humans with Parkinsonism”	2015	Proc. Natl. Acad. Sci. USA
Masuda-Suzukake	“Pathological Alpha-Synuclein Propagates through Neural Networks”	2014	Acta Neuropathol. Commun.
Sacino	“Brain Injection of Alpha-Synuclein Induces Multiple Proteinopathies, Gliosis, and a Neuronal Injury Marker”	2014	J. Neurosci.
Kovacs	“Intracellular Processing of Disease-Associated *α*-Synuclein in the Human Brain Suggests Prion-Like Cell-to-Cell Spread”	2014	Neurobiol. Dis.
Recasens	“Lewy Body Extracts from Parkinson Disease Brains Trigger *α*-Synuclein Pathology and Neurodegeneration in Mice and Monkeys”	2014	Ann. Neurol.
Ulusoy	“Caudo-Rostral Brain Spreading of *α*-Synuclein through Vagal Connections”	2013	EMBO Mol. Med.
Masuda-Suzukake	“Prion-Like Spreading of Pathological *α*-Synuclein in Brain”	2013	Brain
Angot	“Alpha-Synuclein Cell-to-Cell Transfer and Seeding in Grafted Dopaminergic Neurons *In Vivo*”	2012	PLoS One
Luk	“Intracerebral Inoculation of Pathological Alpha-Synuclein Initiates a Rapidly Progressive Neurodegenerative Alpha-Synucleinopathy in Mice”	2012	J. Exp. Med.
Luk	“Pathological Alpha-Synuclein Transmission Initiates Parkinson-Like Neurodegeneration in Nontransgenic Mice”	2012	Science
Kordower	“Transfer of Host-Derived Alpha Synuclein to Grafted Dopaminergic Neurons in Rat”	2011	Neurobiol. Dis.
Hansen	“Alpha-Synuclein Propagates from Mouse Brain to Grafted Dopaminergic Neurons and Seeds Aggregation in Cultured Human Cells”	2011	J. Clin. Invest.
Danzer	“Seeding Induced by Alpha-Synuclein Oligomers Provides Evidence for Spreading of Alpha-Synuclein Pathology”	2009	J. Neurochem.
Kordower	“Transplanted Dopaminergic Neurons Develop PD Pathologic Changes: A Second Case Report”	2008	Mov. Disord.
Kordower	“Lewy Body-Like Pathology in Long-Term Embryonic Nigral Transplants in Parkinson's Disease”	2008	Nat. Med.
Li	“Lewy Bodies in Grafted Neurons in Subjects with Parkinson's Disease Suggest Host-to-Graft Disease Propagation”	2008	Nat. Med.

	*Evidences confuting α-synuclein spreading*		
Sumikura	“Distribution of Alpha-Synuclein in the Spinal Cord and Dorsal Root Ganglia in an Autopsy Cohort of Elderly Persons”	2015	Acta Neuropathol. Commun.
Sacino	“Amyloidogenic *α*-Synuclein Seeds Do Not Invariably Induce Rapid, Widespread Pathology in Mice”	2014	Acta Neuropathol.
Halliday	“The Progression of Pathology in Parkinson's Disease”	2010	Ann. N. Y. Acad. Sci.
Jang	“Non-Classical Exocytosis of Alpha-Synuclein Is Sensitive to Folding States and Promoted under Stress Conditions”	2010	J. Neurochem.
Hawkes	“Parkinson's Disease and Aging: Same or Different Process?”	2008	Mov. Disord.
Kalaitzakis	“Controversies over the Staging of Alpha-Synuclein Pathology in Parkinson's Disease”	2008	Acta Neuropathol.
